# Influence of Mast Cells on Dengue Protective Immunity and Immune Pathology

**DOI:** 10.1371/journal.ppat.1003783

**Published:** 2013-12-19

**Authors:** Ashley L. St. John

**Affiliations:** 1 Program in Emerging Infectious Diseases, Duke-National University of Singapore Graduate Medical School, Singapore; 2 Pathology Department, Duke University Medical Center, Durham, North Carolina, United States of America; Columbia University, United States of America

## 1. Mast Cells in Immune Protection and Pathology

Mast cells (MCs) are sentinels for pathogens. They are situated in the skin and mucosae where pathogen encounter is common and surround blood vessels. MCs pre-store vasoactive mediators and cytokines in granules, such as histamine, heparin, proteases, and TNF [1]. Degranulation occurs virtually instantaneously after MC activation by certain pathogen-derived or endogenous products signifying infection or inflammation. While MC activation through pattern recognition receptors, such as TLRs, alone is not usually a sufficient cause for degranulation, degranulation-initiating receptors have been identified for bacterial pathogens (e.g., CD48 for *E. coli, M. tuberculosis*) [2]. Some viruses trigger degranulation, including dengue, through yet-unknown receptors [2]. MCs also express Fc receptors for binding antibodies, such as FcεR1 that binds IgE. MC binding of IgE, or sensitization, heightens degranulation responses to IgE-specific antigens when antibodies are present, as occurs during reinfection [1]. MCs also *de novo* synthesize products, including leukotrienes, prostaglandins, cytokines, and chemokines [1].

MCs promote protective immunity against pathogens but, paradoxically, they are best characterized for contributing to pathology when they are chronically activated (as in the context of allergy) or when substantial acute activation causes systemic excess of their products (e.g., during anaphylaxis, which can lead to shock). Thus, when misdirected, such as to environmental antigens, or when excessive, prolonged, or systemic, MC responses can harm the host, causing vascular leakage or tissue damage. The potential for MCs to promote a spectrum of disease outcomes, ranging from protective immunity to immune pathology, is apparent during bacterial peritonitis, where MCs either protect from or promote death, depending on the severity of the experimental model [3]. To date, we know little about the role of MCs during acute viral infections [1]. However, evidence has emerged that MCs significantly influence immunity and pathogenesis during dengue virus (DENV) infection.

## 2. Immunosurveillance for Dengue Virus by Mast Cells

DENV, a positive-sense single-stranded RNA virus, is a member of the Flavivirus family and an arboviral pathogen with substantial worldwide burden. All four serotypes of DENV can cause disease ranging from mild febrile illness (dengue fever) to life-threatening complications (dengue hemorrhagic fever, DHF), characterized by severe vascular pathology [4]. When infection begins, mosquitoes inject virus while probing extensively under the skin in search of a blood meal. They also inject saliva, damage tissues, and break capillaries [5]. DENV infects Langerhans cells, and may also infect other DC and monocyte subtypes [6]. Like Langerhans cells, MCs are tissue-resident and encounter DENV in the earliest moments of infection, yet MC interactions with DENV are markedly different from those of other antigen-presenting cell (APC) types that are targets of infection. MCs degranulate within minutes of exposure to DENV, followed by *de novo* cytokine production in the subsequent hours [7]. Detection of the MC-specific and granule-associated product chymase in the serum of DENV patients with acute infection also demonstrates MC degranulation occurs *in vivo* during the course of clinically significant infections [8]. Serum virus titers were lowest in DHF patients with high chymase levels, suggesting that MC activation might limit infection in humans [8]. Studies using MC-deficient mice have shown that MC activation during DENV infection dramatically limits infection at the initial skin infection site and in draining lymph nodes (LNs), secondary sites of infection [7]. Since they are distributed throughout the skin where virus inoculation first occurs, and pre-store many immune-modulatory and vasoactive mediators that are released within minutes of DENV exposure, MCs should be the first cells capable of detecting DENV and raising the initial alarm of infection.

**Figure 1:**
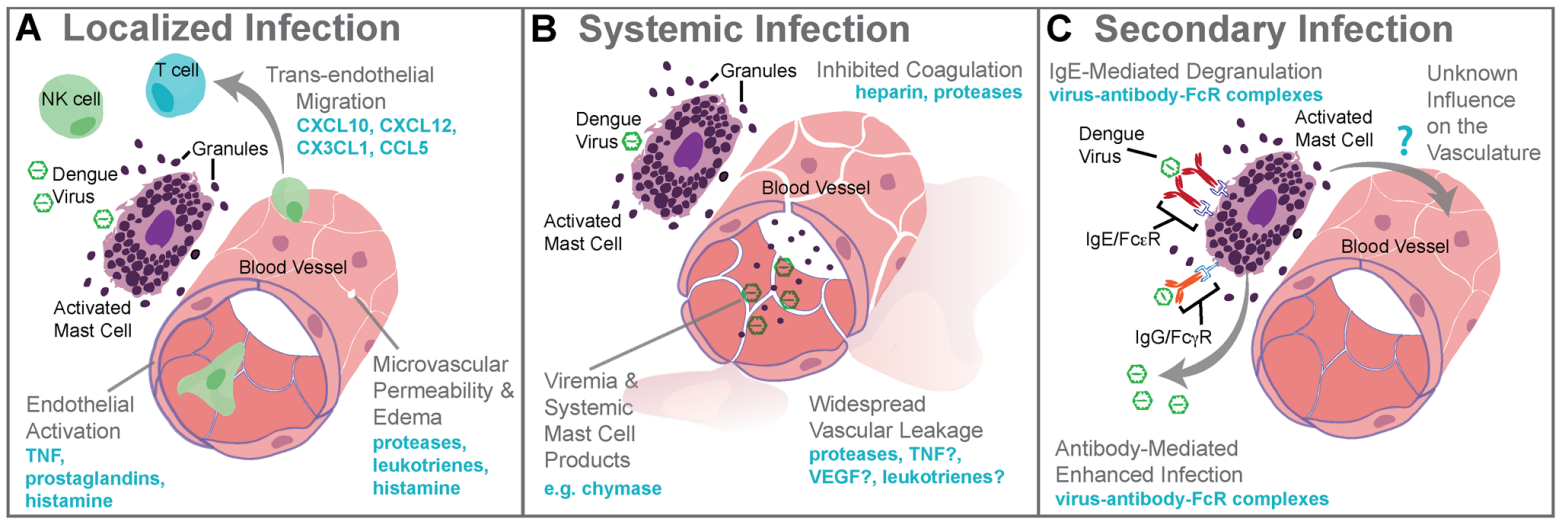
Mast cell–augmented immune responses to dengue virus infection. (A) During localized infection of the skin, DENV triggers degranulation of MCs and release of *de novo* synthesized inflammatory mediators. MC-derived mediators, including proteases, leukotrienes, and histamine, promote edema within the site of infection as a result of increased microvascular permeability. MC products also stimulate the rolling of leukocytes along areas of activated endothelium and chemokines direct the recruitment of cytotoxic cells such as NK cells, NKT cells, and T cells into the infection site. The antiviral inflammatory program promoted by MCs at the site of infection aids viral clearance and limits spread beyond the site of initial infection to lymph nodes. (B) Systemic infection with DENV is characterized by widespread vascular leakage and virema coincides with elevated levels of MC products, such as the protease chymase, in the serum. Many MC products are vasoactive, including leukotrienes, TNF, VEGF, and others, and these are likely to act together to enhance the vascular leakage that occurs during viremia. (C) During secondary infection, pre-formed antibodies are hypothesized to cause ADE when they are non-neutralizing. For mast cells, ADE is also possible as a result of uptake of antibody virus complexes through the FcγR. MC degranulation responses can also be enhanced through crosslinking of FcεRs when bound to DENV-specific IgE. Although the influence of DENV-specific antibodies acting through MCs has not fully been investigated, augmented MC activation during secondary infection should also enhance immune responses, presumably including immune-mediated vascular injury.

Localized MC responses in the skin promote vasodilation, endothelial activation, and cellular recruitment to aid pathogen clearance [1] (Figure 1A). During DENV infection, a virus-specific MC-dependent immune program is dominated by the recruitment of NK and T cells, with NKT cells particularly enriched in DENV-infected skin [7] (Figure 1A). Depletion studies in mice showed that NK1.1^+^ cells, like MCs, promote DENV clearance in the site of infection and also limit spread to LNs [8]. In humans, activated NK cells have also been associated with mild clinical disease [1]. In other infections, MCs promote LN swelling, antigen presentation, DC recruitment, and other processes [1], [2], so there may be additional ways that the ability of MCs to augment immune responses facilitates DENV clearance.

## 3. Mechanisms of Dengue-Induced Mast Cell Activation

Surprisingly, MCs are very resistant to infection by DENV. *In vitro* doses of virus that would cause permissive cell types to become 100% infected, in comparison, infect only ∼3% of MCs [7]. However, evidence suggests that some internalization of DENV occurs by MCs. The replication intermediate, dsRNA, can be formed in MCs, activating intracellular cytosolic sensors RIG-I and MDA-5 and resulting in production of certain cytokines and chemokines [7]. This observation suggests that replication is initiated in MCs but fails to produce functional virus particles. TLR3 also contributed to DENV-induced TNF production by MCs [7]. Importantly, none of these receptors appears to influence DENV-induced MC degranulation, since siRNA targeting of the receptors did not reduce degranulation responses in spite of effectively dampening cytokine production. UV-inactivated DENV is also sufficient to provoke MC degranulation [7]. These two observations emphasize that productive viral infection is not required for MC degranulation and suggests that degranulation in response to DENV is probably dependent on an unidentified cell surface receptor.

MCs have a unique ability to detect intact viral particles independent of infection, but during infection *in vivo*, additional activating stimuli may also potentiate MC-driven early innate immune responses to DENV. For example, endogenous products such as complement split products can induce MC degranulation through complement receptors [1], [9]. MCs also are activated by mosquito saliva [10], which is co-injected with virus during natural-route arboviral infections. MC histamine promotes the characteristic wheal and flare reaction observed at the site of a mosquito bite [11]. Finally, since MCs express multiple Fc receptors, the presence of preexisting antibodies also would be likely to modulate immunity [1], as discussed below.

## 4. Mast Cell Contributions to Dengue Vascular Pathology

MC responses that are productive for clearing localized infection are not always beneficial on a systemic scale. In humans, DENV usually achieves systemic infection within days of a mosquito bite [12], and at that time the patient experiences viremia and fever [4]. There is an increased likelihood of vascular pathology such as bruising of the skin, even in mild DENV cases, but in some patients vascular pathology becomes severe, resulting in vascular leakage, pooling of plasma within tissues, and, potentially, hypovolemic shock [13]. These symptoms are also associated with MC activation in independent clinical contexts, raising the question of whether MCs also contribute to vascular pathology during DENV infection. Currently, the mechanisms underlying DENV-induced pathophysiological changes are controversial; however, vascular leakage is largely attributed to immune pathology, as opposed to direct infection of endothelial cells [13]. Several mechanisms have been proposed to explain immune pathogenesis during DENV infection, and one that has the potential to occur during primary infection is “cytokine storm” [14]. Infected cells, such as monocytes, are a major source of cytokines, (e.g., TNF, which can promote vascular leakage). In immunocompromised mice, TNF significantly contributed to early death due to dengue [15]. In addition to cytokines from infected cells, healthy immune cells are likely to participate in the escalated production of inflammatory mediators *in vivo*. MCs also release vasoactive cytokines and their granules contain proteases, which induce break down of endothelial tight junctions and reduce blood clotting [11] (Figure 1B). MC-deficient mice given systemic DENV infections had greatly reduced vascular leakage compared to MC-sufficient controls [8]. In support of a MC-dependent component of DENV-induced vascular leakage, drugs that stabilize MCs (e.g., cromolyn and/or ketotifen) limited leakage in the wild type and immune-compromised mouse models of DENV [8]. The leukotriene receptor antagonist, montelukast, was also effective [8]. These findings raise the possibility of using MC-stabilizing compounds to treat DENV pathology in humans.

## 5. Dengue Immunological Memory and Mast Cells

**Figure 2:**
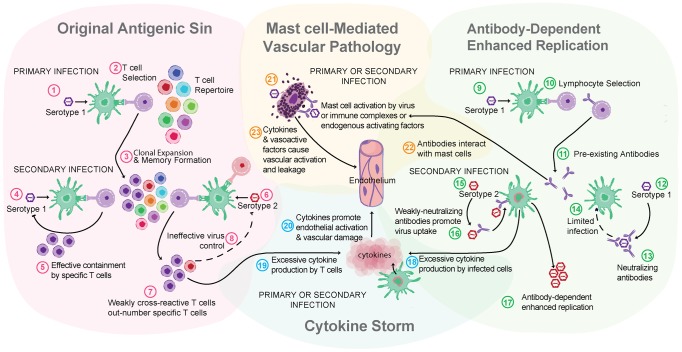
Multiple theories of dengue immune pathogenesis. “Original antigenic sin” has the potential to occur during a DENV secondary infection with a heterologous serotype of DENV. For example, this begins when (1) primary infection occurs with Serotype 1 of DENV, resulting in adaptive immune responses where (2) Serotype 1–specific T cells are selected, activated, and (3) clonally expanded to combat infection. During the resolution of primary infection, memory Serotype 1–specific T cells are formed and are retained in higher frequency in the T cell repertoire than other T naïve cells. (4) A secondary challenge Serotype 1 would evoke a memory recall response and (5) effective containment of infection by highly specific T cells. (6) A secondary challenge with a heterologous strain, Serotype 2, has the potential to reactivate memory T cells that are of greater specificity for Serotype 1 than for Serotype 2. (7) These memory Serotype 1–specific T cells outcompete naïve T cells that would be more specific for Serotype 2, resulting in an expanded memory T cell pool that is low specificity for Serotype 2 and (8) poor viral clearance *in vivo*. Antibody-dependent enhanced replication also has the potential to occur during a secondary, heterologous infection. During primary infection (9), B cell selection occurs, promoting Serotype 1–specific antibody production. (10) These preexisting antibodies are present during the secondary challenge. (12) If the secondary challenge is with Serotype 1 again, (13) antibody-mediated neutralization of DENV occurs, (14) limiting infection. (15) If the secondary challenge is heterologous, as with Serotype 2, antibody specificity may be low (16) and weakly neutralizing antibodies can promote Fc receptor–mediated uptake of virus-antibody complexes. (17) Increased uptake of virus into the cell without efficient antibody-mediated neutralization results in production of higher viral titers and increases activation of pro-inflammatory intracellular signaling pathways. (18) Cytokine storm can occur during either primary or secondary infection when infected cells produce high levels of cytokines or (19) may also be derived from noninfected immune cells, such as activated T cells. (20) Cytokines act directly on the host vasculature and promote vascular leakage when they reach pathological levels. (21) MCs also release cytokines and additional *de novo* synthesized and pre-stored vasoactive mediators when they are activated by DENV. (22) Prior to secondary infection, MCs may also be sensitized by binding DENV-specific antibodies, which can also mediate MC activation in response to DENV. (23) MC-derived mediators act directly on the host vasculature to promote vascular leakage.

Particularly in locations where DENV is endemic, the risk of acquiring a secondary infection with another serotype of DENV is high. In contrast to a secondary infection with the original infecting serotype of DENV, which does not cause symptomatic infection, a secondary heterologous infection (or maternal transmission of antibodies to infants) is a risk factor for severe DENV disease [4], [14]. Two other major theories of dengue pathogenesis that have found experimental support are relevant in the context of secondary infection: original antigenic sin, which describes the potential of low-specificity T cell responses to a secondary heterologous challenge to mediate pathology, and antibody-enhanced infection (ADE), where binding of DENV/non-neutralizing antibody complexes by cells promotes DENV uptake and replication [15]–[17] (Figure 2).

MCs are also effector cells of immunological memory since they are able to bind multiple subclasses of antibodies through Fc receptors. They have specialized responses to unique stimuli and although there are critical distinctions between their responses to a pathogen challenge and a challenge with an innocuous antigen, in the context of secondary infection to DENV, viral particles may also act similarly to an antigen challenge by promoting Fc receptor aggregation on MCs. It has been shown that DENV-induced activation of MCs sensitized with post-immune serum can trigger degranulation [18] (Figure 1C). Antibody-mediated interactions between MCs and DENV are dramatically different from the context of naïve exposure, but the relative contributions of various subclasses of antibodies have not been fully investigated. First, MC exposure to DENV immune complexes with IgG promotes infection, similar to ADE responses that occur with monocytes [19] (Figure 1C). Whether this increased infection burden is due to an altered endocytic pathway and infection route, to increased viral uptake, or both is not clear. Second, MCs have enhanced sensitivity to DENV-induced degranulation in the presence of DENV-specific IgE, so lower concentrations of virus are required to elicit detectable responses [7], [18] (Figure 1C). In DENV patients, IgE has been associated with developing DHF [20], and MC activation levels (measured using the biomarker chymase) are also higher in DHF patients experiencing secondary infection compared to primary infection [8]. These findings suggest that, in addition to the ADE mechanism of achieving higher virus titers during heterologous secondary infection in humans, antibodies can enhance MC release of vasoactive inflammatory products (Figure 1C, Figure 2).

## Conclusions

Localized responses of MC to DENV in the skin are protective by promoting vasodilation and cellular recruitment, which aid viral clearance. In contrast, MC-induced vascular leakage on a systemic level can contribute to DENV pathogenesis and vascular leakage, both during primary infections and due to antibody-enhanced MC responses and/or infection during secondary infection.

## References

[1] Abraham SN, St. John AL (2010) Mast cell-orchestrated immunity to pathogens. Nat Rev Immunol 10: 440–452. 20498670 10.1038/nri2782 PMC4469150

[2] St. John AL, Abraham SN (2013) Innate immunity and its regulation by mast cells. J Immunol 190: 4458–4463. 23606723 10.4049/jimmunol.1203420 PMC3645001

[3] Piliponsky AM, Chen CC, Grimbaldeston MA, Burns-Guydish SM, Hardy J, et al (2010) Mast cell-derived TNF can exacerbate mortality during severe bacterial infections in C57BL/6-KitW-sh/W-sh mice. Am J Pathol 176: 926–938. 20035049 10.2353/ajpath.2010.090342 PMC2808097

[4] Halstead SB (2007) Dengue. Lancet 370: 1644–1652. 17993365 10.1016/S0140-6736(07)61687-0

[5] Choumet V, Attout T, Chartier L, Khun H, Sautereau J, et al (2012) Visualizing non infectious and infectious *Anopheles gambiae* blood feedings in naive and saliva-immunized mice. PLoS ONE 7: e50464. doi:10.1371/journal.pone.0050464. 23272060 10.1371/journal.pone.0050464 PMC3521732

[6] Wu SJ, Grouard-Vogel G, Sun W, Mascola JR, Brachtel E, et al (2000) Human skin Langerhans cells are targets of dengue virus infection. Nat Med 6: 816–820. 10888933 10.1038/77553

[7] St. John AL, Rathore AP, Yap H, Ng ML, Metcalfe DD, et al (2011) Immune surveillance by mast cells during dengue infection promotes natural killer (NK) and NKT-cell recruitment and viral clearance. Proc Natl Acad Sci U S A 108: 9190–9195. 21576486 10.1073/pnas.1105079108 PMC3107258

[8] St. John AL, Rathore AP, Raghavan B, Ng ML, Abraham SN (2013) Contributions of mast cells and vasoactive products, leukotrienes and chymase, to dengue virus-induced vascular leakage. eLife 2: e00481. 23638300 10.7554/eLife.00481 PMC3639510

[9] Nilsson G, Johnell M, Hammer CH, Tiffany HL, Nilsson K, et al (1996) C3a and C5a are chemotaxins for human mast cells and act through distinct receptors via a pertussis toxin-sensitive signal transduction pathway. J Immunol 157: 1693–1698. 8759757

[10] Demeure CE, Brahimi K, Hacini F, Marchand F, Peronet R, et al (2005) Anopheles mosquito bites activate cutaneous mast cells leading to a local inflammatory response and lymph node hyperplasia. J Immunol 174: 3932–3940. 15778349 10.4049/jimmunol.174.7.3932

[11] Kunder CA, St. John AL, Abraham SN (2011) Mast cell modulation of the vascular and lymphatic endothelium. Blood 118: 5383–5393. 21908429 10.1182/blood-2011-07-358432 PMC3217344

[12] Chan M, Johansson MA (2012) The incubation periods of dengue viruses. PLoS ONE 7: e50972. doi:10.1371/journal.pone.0050972. 23226436 10.1371/journal.pone.0050972 PMC3511440

[13] St. John AL, Abraham SN, Gubler DJ (2013) Barriers to preclinical investigations of anti-dengue immunity and dengue pathogenesis. Nat Rev Microbiol 11: 420–426. 23652323 10.1038/nrmicro3030

[14] Rothman AL (2011) Immunity to dengue virus: a tale of original antigenic sin and tropical cytokine storms. Nat Rev Immunol 11: 532–543. 21760609 10.1038/nri3014

[15] Zellweger RM, Prestwood TR, Shresta S (2010) Enhanced infection of liver sinusoidal endothelial cells in a mouse model of antibody-induced severe dengue disease. Cell Host Microbe 7: 128–139. 20153282 10.1016/j.chom.2010.01.004 PMC2824513

[16] Halstead SB, O'Rourke EJ (1977) Antibody-enhanced dengue virus infection in primate leukocytes. Nature 265: 739–741. 404559 10.1038/265739a0

[17] Balsitis SJ, Williams KL, Lachica R, Flores D, Kyle JL, et al (2010) Lethal antibody enhancement of dengue disease in mice is prevented by Fc modification. PLoS Pathog 6: e1000790. doi:10.1371/journal.ppat.1000790. 20168989 10.1371/journal.ppat.1000790 PMC2820409

[18] Sanchez LF, Hotta H, Hotta S, Homma M (1986) Degranulation and histamine release from murine mast cells sensitized with dengue virus-immune sera. Microbiol Immunol 30: 753–759. 2431252 10.1111/j.1348-0421.1986.tb03002.x

[19] King CA, Marshall JS, Alshurafa H, Anderson R (2000) Release of vasoactive cytokines by antibody-enhanced dengue virus infection of a human mast cell/basophil line. J Virol 74: 7146–7150. 10888655 10.1128/jvi.74.15.7146-7150.2000 PMC112233

[20] Koraka P, Murgue B, Deparis X, Setiati TE, Suharti C, et al (2003) Elevated levels of total and dengue virus-specific immunoglobulin E in patients with varying disease severity. J Med Virol 70: 91–98. 12629649 10.1002/jmv.10358

